# Multiple Delivery of siRNA against Endoglin into Murine Mammary Adenocarcinoma Prevents Angiogenesis and Delays Tumor Growth

**DOI:** 10.1371/journal.pone.0058723

**Published:** 2013-03-05

**Authors:** Tanja Dolinsek, Bostjan Markelc, Gregor Sersa, Andrej Coer, Monika Stimac, Jaka Lavrencak, Andreja Brozic, Simona Kranjc, Maja Cemazar

**Affiliations:** 1 Department of Experimental Oncology, Institute of Oncology Ljubljana, Ljubljana, Slovenia; 2 Department of Natural and Medical Subjects, Faculty of Health Sciences, University of Primorska, Izola, Slovenia; 3 Department of Cytopathology, Institute of Oncology Ljubljana, Ljubljana, Slovenia; Duke University Medical Center, United States of America

## Abstract

Endoglin is a transforming growth factor-β (TGF- β) co-receptor that participates in the activation of a signaling pathway that mediates endothelial cell proliferation and migration in angiogenic tumor vasculature. Therefore, silencing of endoglin expression is an attractive approach for antiangiogenic therapy of tumors. The aim of our study was to evaluate the therapeutic potential of small interfering RNA (siRNA) molecules against endoglin *in vitro* and *in vivo*. Therapeutic potential *in vitro* was assessed in human and murine endothelial cells (HMEC-1, 2H11) by determining endoglin expression level, cell proliferation and tube formation. *In vivo*, the therapeutic potential of siRNA molecules was evaluated in TS/A mammary adenocarcinoma growing in BALB/c mice. Results of our study showed that siRNA molecules against endoglin have a good antiangiogenic therapeutic potential *in vitro*, as expression of endoglin mRNA and protein levels in mouse and human microvascular endothelial cells after lipofection were efficiently reduced, which resulted in the inhibition of endothelial cell proliferation and tube formation. *In vivo*, silencing of endoglin with triple electrotransfer of siRNA molecules into TS/A mammary adenocarcinoma also significantly reduced the mRNA levels, number of tumor blood vessels and the growth of tumors. The obtained results demonstrate that silencing of endoglin is a promising antiangiogenic therapy of tumors that could not be used as single treatment, but as an adjunct to the established cytotoxic treatment approaches.

## Introduction

Antiangiogenic therapies of cancer represent a promising approach to the conventional systemic treatments of cancer. Namely, tumors depend on angiogenesis to grow beyond a few mm^3^, as they are unable to grow in a lack of oxygen and nutrients. Endothelial cells in angiogenic tumor vasculature proliferate much faster than in normal vasculature, therefore they represent an ideal therapeutic target. The fast proliferation is also due to increased expression of certain endothelial cell markers [1], [2]. One of these markers is endoglin (CD105), a TGF-β co-receptor, which participates in the activation of a complex signaling pathway that mediates cell proliferation, migration and adhesion [3], [4]. Endoglin expression is highly elevated in proliferating vascular endothelial cells within and in the surrounding of the tumors [5]–[7]. Due to the increased expression of endoglin in angiogenic endothelium, antibodies targeting endoglin have already been used for tumor imaging [8]–[11] and endoglin was proposed to be used as prognostic tumor marker [12]–[14]. Moreover, several *in vitro* studies showed that different anti-endoglin antibodies inhibit proliferation and tube formation of human endothelial cells [15]–[17], which was a strong experimental support for the usage of anti-endoglin antibodies for vascular targeted therapy *in vivo*. Most of these studies were done in immunodeficient SCID mice bearing human MCF7 mammary carcinoma. When repetitive treatments with toxin-conjugated or radiolabelled anti-endoglin antibodies were performed, a long-lasting regression of tumor growth was obtained, whereas naked antibodies had less pronounced effect on tumor growth [18]–[21]. Anti-tumor effectiveness and anti-metastatic activity of naked anti-endoglin antibodies were also reported in immunocompetent BALB/c mice bearing 4T1 mammary carcinoma or colon-26 colon carcinoma [22]–[24].

Silencing of endoglin mRNA by RNA interference is another attractive approach for endoglin targeting. This approach has been studied *in vitro* and demonstrated that silencing of endoglin reduces the proliferation of mouse embryonic endothelial cells [25]. However, the effect of silencing of endoglin on *in vitro* antiangiogenic potential and *in vivo* tumor growth reduction has not been studied yet. Therefore, the aim of our study was to evaluate the therapeutic potential of siRNA molecules against endoglin *in vitro* and *in vivo*. Firstly, the expression level of endoglin in endothelial cell lines *in vitro* and mammary tumors *in vivo* after endoglin silencing was assessed. Secondly, the effect of silencing of endoglin on proliferation and tube formation of endothelial cells was determined *in vitro*. Finally, the therapeutic potential of siRNA molecules against endoglin was investigated *in vivo* in BALB/c mice bearing TS/A mammary adenocarcinoma.

## Results

### Endoglin expression in endothelial cells, tumor cells and tumors

Endoglin expression in HMEC-1 human endothelial cells, 2H11 murine endothelial cells, TS/A tumor cells and TS/A subcutaneous tumors from BALB/c mice was determined with quantitative real time polymerase chain reaction (qRT-PCR). HMEC-1 and 2H11 cells expressed high levels of endoglin. In TS/A tumor cells the expression of endoglin was very low. In contrast, TS/A tumors induced in BALB/c mice express higher levels of endoglin, indicating that the elevated endoglin level originate from the endothelial cells present in the tumor blood vessels rather than from the tumor cells themselves (Table 1).

**Table 1:** Expression of endoglin in endothelial cells, tumor cells and tumors, expressed as threshold cycle values (Ct) obtained by qRT-PCR in comparison to reference gene.

	Ct (endoglin)	Ct (reference gene)
HMEC-1 cells	20.7	21.5 (human RNA polymerase II)
2H11 cells	25.2	8.5 (murine 18S ribosomal RNA)
TS/A cells	37.6	9.5 (murine 18S ribosomal RNA)
TS/A tumor	26.7	9.1 (murine 18S ribosomal RNA)

### Endoglin expression was reduced after the transfection of endothelial cells with siRNA molecules

**Figure 1:**
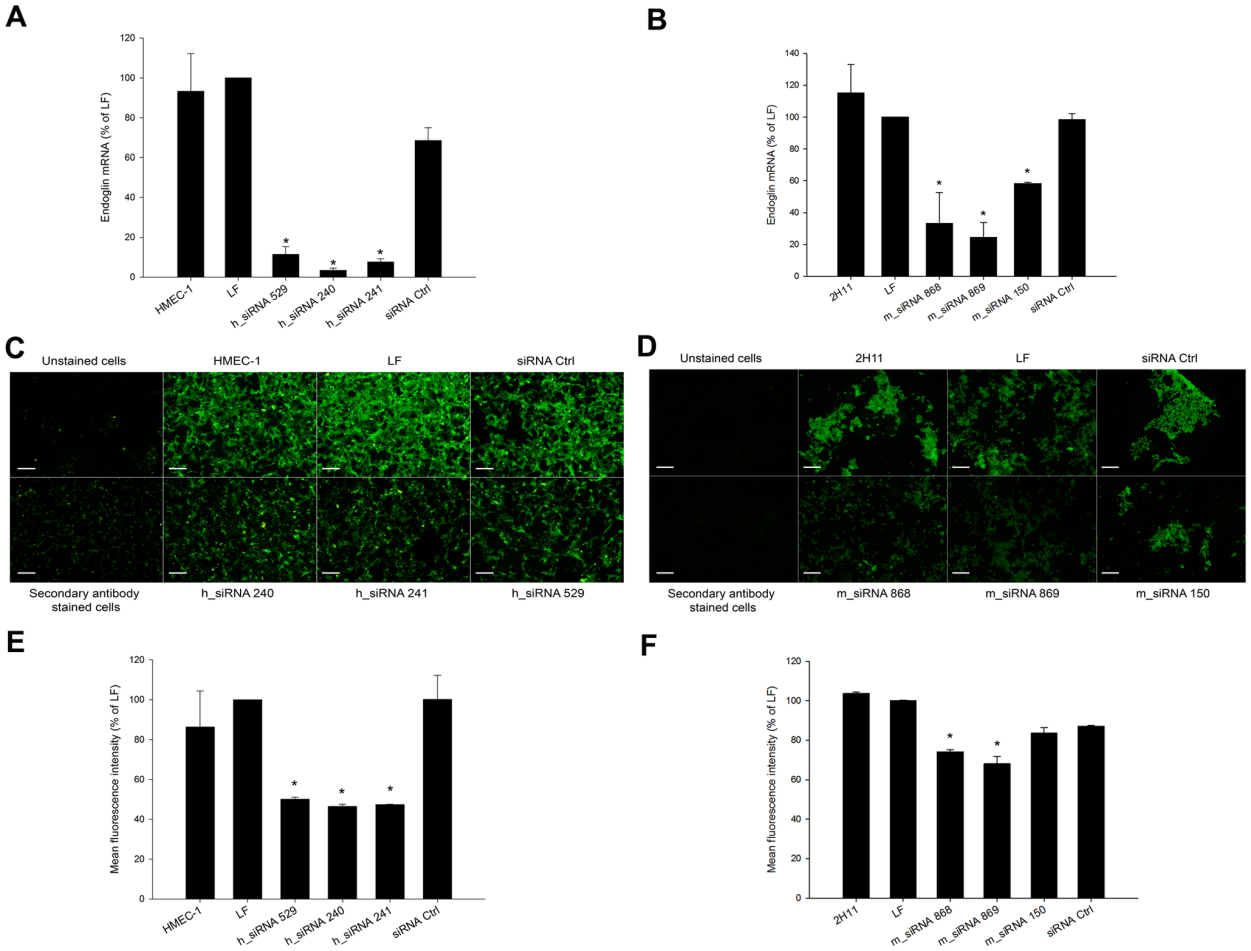
Transfection of endothelial cells with siRNA against endoglin resulted in reduced mRNA and protein levels. (A) qRT-PCR analysis of endoglin mRNA level after the transfection of endothelial cells HMEC-1 with 3 different siRNA molecules (h_siRNA 529, h_siRNA 240, h_siRNA 241). (B) qRT-PCR analysis of endoglin mRNA level after the transfection of endothelial cells 2H11 with 3 different siRNA molecules (m_siRNA 868, m_siRNA 869, m_siRNA 150). mRNA level of endoglin in cells treated with siRNA molecules was normalized to the mRNA level of endoglin in cells treated with Lipofectamine RNAiMAX only (% of LF); *P<0.05 vs. untreated control cells (HMEC-1 or 2H11), LF and negative control siRNA (siRNA Ctrl). Data represent arithmetic mean±SEM. (C) Immunofluorescence staining of endoglin after the transfection of endothelial cells HMEC-1 with 3 different siRNA molecules (h_siRNA 529, h_siRNA 240, h_siRNA 241). (D) Immunofluorescence staining of endoglin after the transfection of endothelial cells 2H11 with 3 different siRNA molecules (m_siRNA 868, m_siRNA 869, m_siRNA 150). (E) Mean fluorescence intensity of HMEC cells stained for endoglin determined with flow cytometry analysis after the transfection of cells with 3 different siRNA molecules (h_siRNA 529, h_siRNA 240, h_siRNA 241). (F) Mean fluorescence intensity of 2H11 cells stained for endoglin determined with flow cytometry analysis after the transfection of cells with 3 different siRNA molecules (m_siRNA 868, m_siRNA 869, m_siRNA 150). Values are normalized to Lipofectamine RNAiMAX group (% of LF). *P<0.05 vs. untreated control cells (HMEC-1, 2H11), LF and siRNA Ctrl.

Two days after the transfection of HMEC-1 and 2H11 cells with siRNA molecules targeting different sections of coding sequences of endoglin, the endoglin mRNA level in these cells was statistically significantly lower in comparison to endoglin mRNA level of cells treated with Lipofectamine RNAiMAX only and negative control siRNA. In human HMEC-1 endothelial cells, all three tested siRNA molecules (h_siRNA 529, h_siRNA 240, h_siRNA 241) reduced the level of endoglin mRNA for the approximately same level, ranging from 88 to 96% (Figure 1A). In murine endothelial cell line, two tested siRNA molecules (m_siRNA 868, m_siRNA 869) were equally effective in reducing the level of endoglin mRNA (Figure 1B), while m_siRNA 150 only marginally reduced endoglin mRNA level.

Besides endoglin mRNA level, the silencing effect of siRNA molecules on endoglin expression was also determined by immunofluorescence staining of endoglin and flow cytometry analysis of endoglin positive cells. In HMEC-1 cells, silencing of endoglin with all three tested siRNAs (h_siRNA 529, h_siRNA 240, h_siRNA 241) was demonstrated by a reduced immunofluorescence staining for endoglin (Figure 1C). In 2H11 cells, immunofluorescence staining of 2H11 cells showed that m_siRNA 868 and m_siRNA 869 reduce amount of endoglin to a greater extent as m_siRNA 150, as was also demonstrated at the mRNA level by qRT-PCR at the mRNA level (Figure 1D).

Flow cytometry analysis of endoglin positive endothelial cells confirmed the results obtained by immunofluorescence staining. Mean fluorescence intensity of HMEC-1 cells was statistically significantly decreased for∼50% after transfection with all 3 siRNAs compared to cells that were treated with Lipofectamine RNAiMAX only (Figure;1E). On the other hand, mean fluorescence intensity of 2H11 cells was statistically significantly reduced for m_siRNA 868 and m_siRNA 869 (for∼25%), while m_siRNA 150 had minor effect on endoglin protein level as already demonstrated by qRT-PCR and immunofluorescence staining (Figure 1F).

### Proliferation of endothelial cells was reduced after the transfection with siRNA molecules against endoglin

**Figure 2:**
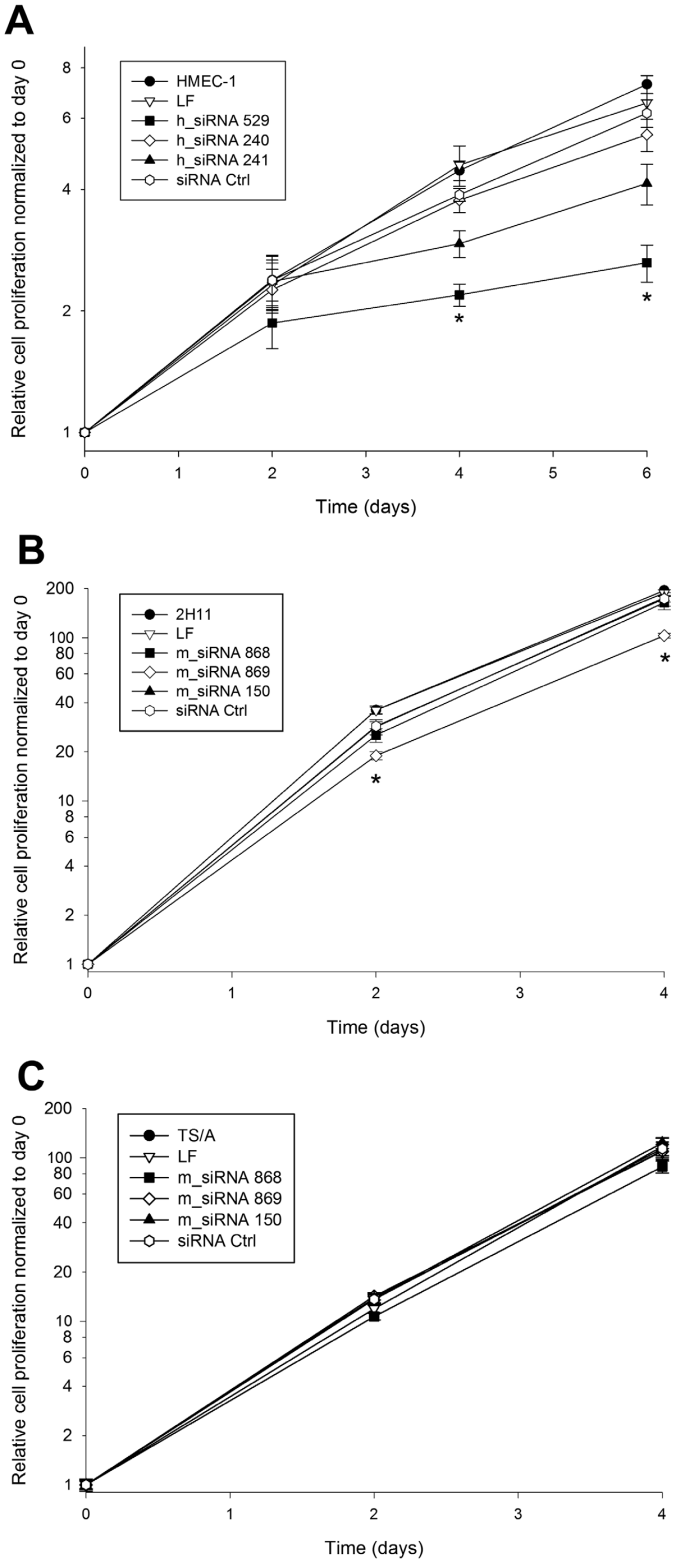
Effect of siRNAs against endoglin on cell proliferation. (A) Proliferation of HMEC-1 cells after the transfection with Lipofectamine RNAiMAX only (LF), h_siRNA 529, h_siRNA 240, h_siRNA 241 and negative control siRNA (siRNA Ctrl). (B) Proliferation of 2H11 cells after the transfection with Lipofectamine RNAiMAX only (LF), m_siRNA 868, m_siRNA 869, m_siRNA 150 and negative control siRNA (siRNA Ctrl). (C) Proliferation of TS/A cells after the transfection with Lipofectamine RNAiMAX only (LF), m_siRNA 868, m_siRNA 869, m_siRNA 150 and negative control siRNA (siRNA Ctrl). Proliferation of cells in each experimental group was normalized to day 0. *P<0.05 vs. untreated control cells (HMEC-1, 2H11 or TS/A), LF and siRNA Ctrl. Note that due to the slower growth of HMEC-1 cells, the y-axis has different range.

The inhibitory effect of silencing of endoglin with different siRNA molecules on proliferation of HMEC-1 (doubling time: 55.0 h) and 2H11 cells (doubling time: 18.6 h) with high endoglin expression was followed for 4–6 days. In HMEC-1 cells, the transfection with h_siRNA 529 resulted in a statistically significant decrease of cell proliferation for∼60% at day 6 after the transfection in comparison to the proliferation of HMEC-1 cells treated with Lipofectamine RNAiMAX only. The other 2 siRNA molecules, h_siRNA 240 and h_siRNA 241 also had effect on cell proliferation, but not statistically significant. In addition, siRNA Ctrl did not significantly reduced cell proliferation (Figure 2A). Due to the fast growth of 2H11 cells, the proliferation was followed for 4 days only. M_siRNA 869 reduced cell proliferation for∼45% at day 4 after the transfection in comparison to the proliferation of 2H11 cells treated with Lipofectamine RNAiMAX only. The other 2 siRNA molecules m_siRNA 868 and m_siRNA 150 as well as siRNA Ctrl did not significantly reduce cell proliferation (Figure 2B).

In addition, we demonstrated that the transfection of siRNA molecules targeting murine endoglin does not reduce the proliferation of murine mammary adenocarcinoma cells TS/A, which express very low levels of endoglin mRNA (Figure 2C).

### Tube formation of endothelial cells was reduced after the transfection with siRNA molecules against endoglin

**Figure 3:**
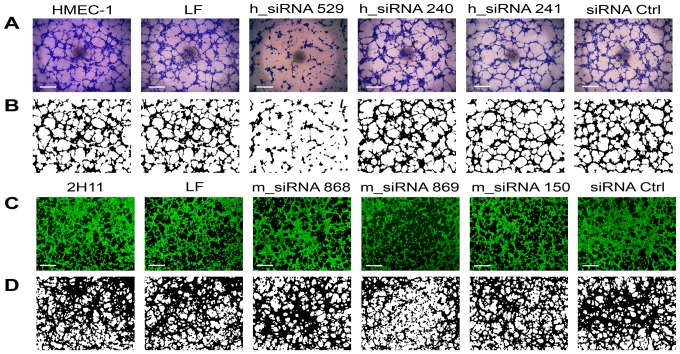
Lipofection of HMEC-1 and 2H11 cells with siRNA against endoglin reduced tube formation. Original images of tubular complexes of HMEC-1 cells (A) and corresponding binary masks (B) 2 days after the transfection with different siRNA molecules: untreated control cells (HMEC-1), cells transfected with Lipofectamine RNAiMAX only (LF), h_siRNA 529, h_siRNA 240, h_siRNA 241 and negative control siRNA (siRNA Ctrl). Original images of tubular complexes of 2H11 cells (C) and corresponding binary masks (D) 2 days after the transfection with different siRNA molecules: untreated control cells (2H11), cells transfected with Lipofectamine RNAiMAX only (LF), m_siRNA 868, m_siRNA 869, m_siRNA 150 and negative control siRNA (siRNA Ctrl). Scale bar: 100 µm.

To determine the antiangiogenic potential of siRNA molecules against endoglin, the endothelial cell tube formation assay (*in vitro* angiogenesis assay) was performed. Two and 3 days after the transfection, h_siRNA 529 inhibited tube formation of HMEC-1 cells. The other 2 siRNA molecules, h_siRNA 240 and h_siRNA 241 as well as siRNA Ctrl did not significantly affect tube formation of HMEC-1 cells, thus confirming the results obtained by cell proliferation (Figure 3A, 3B). The analysis of binary images showed that h_siRNA 529 statistically significantly decreased the total length of tubular complexes, the total size of tubular complexes and the number of junctions at day 2 and 3 post-transfection (Table 2). The decrease was more pronounced at day 2 indicating that the effect of endoglin silencing is short lived. The other two siRNA molecules (h_siRNA 240 and h_siRNA 241) had no significant effect on the measured properties of tubular complexes.

**Table 2:** Total length of tubular complexes, total size of tubular complexes and number of junctions after the transfection of endothelial cells HMEC-1 with 3 different siRNA molecules (h_siRNA 529, h_siRNA 240, h_siRNA 241).

	*Total length of tubular complexes*			*Total size of tubular complexes*			*Number of junctions*		
	Day 1	Day 2	Day 3	Day 1	Day 2	Day 3	Day 1	Day 2	Day 3
HMEC-1	103±2	96±3	104±2	116±4	94±4	96±3	100±3	93±4	106±3
h_siRNA 529	100±2	66±4*	75±7*	106±2	70±3*	70±5*	96±2	61±7*	71±7*
h_siRNA 240	101±2	107±4	113±4	109±5	111±4	114±4	99±1	106±6	111±3
h_siRNA 241	95±4	103±4	109±5	105±10	109±7	112±5	90±2	101±6	108±9
siRNA Ctrl	91±4	103±2	102±5	92±7	107±2	102±6	89±3	100±3	100±6

Data represent arithmetic mean±SEM normalized to cells treated with Lipofectamine RNAiMAX only (% of Lipofectamine RNAiMAX); *P<0.05 vs. untreated control cells (HMEC-1), Lipofectamine RNAiMAX and negative control siRNA (siRNA Ctrl).

Tube formation of 2H11 cells was determined two days after transfection, according to the results obtained in HMEC-1 cells. M_siRNA 869 molecule significantly inhibited tube formation. The other 2 siRNA molecules, m_siRNA 868 and m_siRNA 150 as well as siRNA Ctrl, did not significantly affect tube formation of 2H11 cells, thus confirming the results determined by cell proliferation (Figure 3C, 3D). The analysis of binary images showed that m_siRNA 869 statistically significantly decreased the total length of tubular complexes for∼16%, the total size of tubular complexes for∼13% and the number of junctions for∼22% at the day 2 post-transfection. The other two siRNA molecules (m_siRNA 868 and m_siRNA 150) had no significant effect on the measured properties of tubular complexes.

### Tumor growth and endoglin mRNA level were reduced after the transfection of TS/A tumors with siRNA molecules *in vivo*

**Figure 4:**
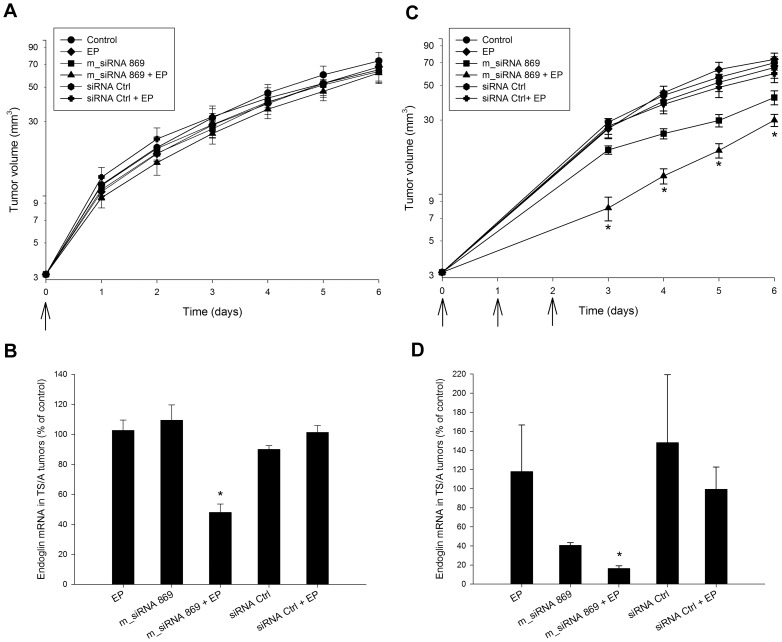
The effect of electrotransfer of siRNA against endoglin in TS/A tumors. (A) Growth of TS/A tumors exposed to a single therapy: intratumoral injection of H_2_O alone (control) or combined with application of electric pulses (EP), intratumoral injection of m_siRNA 869, intratumoral injection of negative control siRNA (siRNA Ctrl) and electrotransfer of m_siRNA 869 (m_siRNA 869+EP) and negative control siRNA (siRNA Ctrl+EP). Arrow represents the day of treatment. (B) qRT-PCR analysis of endoglin mRNA level after a single therapy. (C) Growth of TS/A tumors exposed to repetitive therapies. Arrows represent the days of treatment. (D) qRT-PCR analysis of endoglin mRNA level after repetitive therapies. *P<0.05 vs. all groups.

For *in vivo* experiments on silencing of endoglin in tumor endothelial cells, the murine TS/A mammary adenocarcinoma growing in immunocompetent BALB/c mice was used, since the TS/A cells themselves express almost undetectable levels of endoglin mRNA and our aim was to target support cells within the tumors. In addition, the proliferation of TS/A cells was not altered after transfection with siRNAs against endoglin *in vitro*. Due to the low transfection efficiency of lipofection *in vivo* in tumors, electrotransfer was used for *in vivo* experiments [26]. After a single electrotransfer of m_siRNA 869 in TS/A tumors *in vivo*, no statistically significant reduction of tumor growth was observed (Figure 4A) even though endoglin mRNA level was statistically significantly reduced for ∼50% in comparison to the untreated control tumors at day 2 after the treatment (Figure 4B). After repetitive treatments (triple electrotransfer of m_siRNA 869 in three consecutive days) a statistically significant reduction of tumor growth compared to all other groups was observed in the first 6 days after the beginning of the therapy (Figure 4C) as well as at the level of tumor tripling time (Table S1). However, after this reduction lasting for 6 days, the growth rate of the treated tumors was similar to growth rate of tumors in control group. Other, pertinent control treatments did not affect tumor growth, although in all these treatment groups the tumors received injection of either H_2_O (control and EP group) or m_siRNA Ctrl (m_siRNA Ctrl and m_siRNA Ctrl +EP group) 3 times. The tumors grew at the same growth rate as the tumors, which received single treatments. In addition, the endoglin mRNA level was statistically significantly reduced for more than 80% in comparison to the untreated control tumors at day 2 after the treatments (Figure 4D). Three repetitive intratumoral injections of m_siRNA 869 only also had a minor effect on tumor growth and on endoglin mRNA level, which was reduced for ∼60%, but the difference was not statistically significant. These results, together with the results after single electrotransfer, indicate that the reduction of endoglin mRNA level has to reach at least 60% for an evident tumor response. In addition, the weight of the animals did not change during the observation period indicating that there was no systemic toxicity due to the therapy.

### Number of blood vessels was reduced after the triple transfection of TS/A tumors with siRNA molecules in vivo

**Figure 5:**
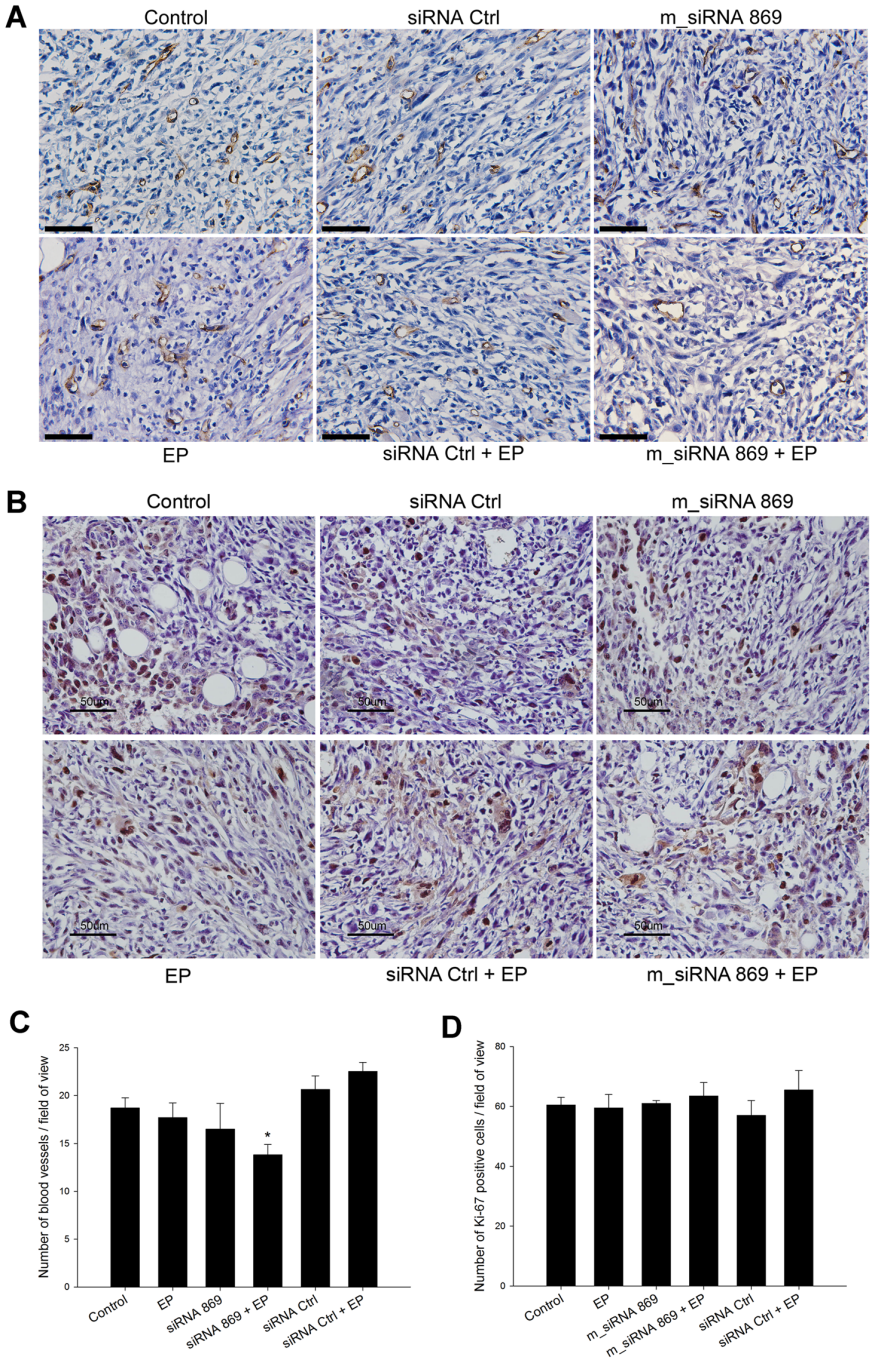
SiRNA against endoglin reduced the number of blood vessels, but not proliferation of tumor cells. Immunohistologically stained tumor sections with anti-CD31 (A) and anti-Ki-67 (B) antibodies after repetitive treatments of TS/A tumors: intratumoral injection of H_2_O alone (control) or combined with application of electric pulses (EP), intratumoral injection of m_siRNA 869, intratumoral injection of negative control siRNA (siRNA Ctrl), electrotransfer of m_siRNA 869 (m_siRNA 869+EP) and negative control siRNA (siRNA Ctrl+EP). Scale bar: 50 μm. (C) Average number of blood vessels smaller than 30 μm per field of view in tumor sections at 30×magnification (D) Average number of Ki-67 positive cells per field of view in tumor sections at 30×magnification. *P<0.05 vs control, siRNA Ctrl and siRNA Ctrl+EP.

Histological analysis was performed 2 days after the triple electrotransfer of m_siRNA 869. In the viable part of tumors there were no morphological changes in endothelial cells or blood vessels wall thickness between different groups. In the group treated with triple electrotransfer of m_siRNA 869 blood vessels with larger lumens were observed. In addition, small blood vessels were not as pronounced as in the control or other groups. Therefore, because endoglin is involved in the activation of endothelial cells, thus sprouting of blood vessels, the CD31 positive blood vessels of the diameter less than 30 μm were counted to determine the antiangiogenic effect of silencing of endoglin. According to the appearance of blood vessels in the histological sections, this cut off point was selected as a representative for tumor capillaries. The triple electrotransfer of m_siRNA 869 significantly reduced the number of blood vessels in tumors. The application of electric pulses alone and the treatment with m_siRNA 869 alone also reduced the number of tumor blood vessels, but to a lesser extent. Treatment of tumors with control siRNA Ctrl alone or in combination with electric pulses did not affect the number of tumor blood vessels (Figure 5A, 5C). Additional staining of tumors for Ki-67 to assess the proliferation of tumor cells after the therapy, demonstrated that the fraction of proliferating tumor cells did not differ between the treatment groups. These results support the results obtained by CD31 staining, demonstrating that the effect on tumor growth is due to the prevention of growth of small vessels and not by direct acting on proliferation of tumor cells (Figure 5B, 5D).

## Discussion

In this study, we showed that siRNA molecules against endoglin have a good antiangiogenic therapeutic potential *in vitro* and *in vivo*. Namely, the expression of endoglin mRNA and protein levels in endothelial cells after the transfection were efficiently reduced, which resulted in an inhibition of endothelial cell proliferation and tube formation *in vitro*. *In vivo*, the silencing effect was evident at the endoglin mRNA level and in the reduced number of tumor blood vessels which resulted in a significantly reduced growth of TS/A tumors after a repetitive electrotransfer of m_siRNA 869.

The overexpression of endoglin in proliferating vascular endothelial cells during tumor angiogenesis and vascular development [3], [5], [27], [28] makes endoglin targeting a promising antiangiogenic therapeutic approach in cancer treatment. Several studies using anti-endoglin antibodies have already been performed. However, to our best knowledge, our study is the first comprehensively evaluating the effect of silencing of endoglin expression with siRNA molecules on antiangiogenic potential *in vitro* and antitumor effectiveness *in vivo*.

In the first part of our study, the antiangiogenic potential of silencing of endoglin *in vitro* in human and murine endothelial cell lines was assessed by cell proliferation and *in vitro* tube formation. Our results showed that h_siRNA 529 as well as m_siRNA 869 are effective therapeutic molecules, as the transfection of endothelial cells with these siRNAs resulted in a reduced endoglin mRNA and protein levels as well as in a decreased cell proliferation and endothelial cell tube formation. Proliferation of endothelial cells was reduced up to ∼60%, which is in agreement with the results obtained in other studies using siRNA against mouse endoglin, or specific antibodies and nanobodies. Lebrin et al. studied the molecular mechanisms by which endoglin regulates endothelial cell functions through TGF-β receptor complex signaling and they demonstrated that silencing of endoglin in the presence of TGF-β reduced the proliferation of mouse embryonic endothelial cells for approximately 60% [25]. Maier et al. demonstrated that anti-endoglin antibody TEC-11 reduced the proliferation of human umbilical vein endothelial cells (HUVEC) and human dermal microvascular endothelial cells (HDMEC) after 3 days of incubation for∼60 and∼75%, respectively [16]. In the study of She et al., 4 different SN6 series antibodies inhibited proliferation of HUVEC cells in the range of 26–55% after 3 days of incubation [17]. Ahmadvand et al., who characterized a new nanobody (single monomeric variable domain of antibodies devoid of light chains) targeting endoglin, also observed∼40% inhibition of proliferation of primary HUVEC cells after 3 days of incubation [15].

*In vitro* tube formation assays provide a rapid assessment of antiangiogenic potential of therapy through measurement of the ability of endothelial cells to form capillary-like tubular structures [29]. In our study, the silencing of endoglin in endothelial cells resulted in antiangiogenic effect, since the transfection of HMEC-1 cells with h_siRNA 529 reduced endothelial cell tube formation *in vitro* for*∼*30%, while transfection of 2H11 cells with m_siRNA 869 resulted in less evident effect, which can be attributed to the very fast growth of 2H11 cells and consequently to the dilution of siRNAs from transfected cells. Similar to our results with siRNA, the inhibitory effect on tube formation was obtained also with anti-endoglin nanobodies [15] and antisense DNA against endoglin mRNA in the presence of TGF-β in primary HUVEC cells [30]. Therefore, our approach using siRNA molecules for silencing of endoglin proved to be effective *in vitro* and comparable to other strategies targeting endoglin with antibodies or DNA oligonucleotides. Based on encouraging *in vitro* results, the *in vivo* study was conducted.

For the *in vivo* experiments, instead of lipofection, clinically validated method electrotransfer was chosen for delivery of m_siRNA 869 into the subcutaneous tumors. Electrotransfer yields higher transfection efficiency compared to lipofection of tumors [26]. In addition, it is effective and safe method for delivery of different molecules to various types of cells *in vitro* and tissues *in vivo* [31]–[33]. It is also in use in the clinical setting combined with chemotherapeutic drugs bleomycin or cisplatin or with plasmid DNA encoding interleukin-12 for treatment of tumors [34]–[37]. Reports on electrotransfer of siRNA to tumors are scarce; so far electrotransfer of siRNAs has been only used for silencing of reporter green fluorescent protein in a stably transfected tumor cells B16F10 and for silencing of vascular endothelial growth factor VEGF and Rho GTPase Rac1 genes [38]–[40]. In the study of Golzio et al., similarly to our results, where mRNA level of endoglin were reduced for 50% after one electrotransfer of m_siRNA 869, the mRNA level of GFP was reduced for 52% [38]. In addition, in that study it was shown that the effect of electrotransfered siRNAs is short lived and lasts less than 5 days [38]. Therefore, to demonstrate the therapeutic potential of siRNA against endoglin *in vivo*, a repetitive (3-times) treatment was utilized and compared to a single treatment. Repetitive approach was used also in other studies on the electrotransfer of therapeutic siRNAs [39], [40]. In our study, a single treatment of tumors with m_siRNA 869 electrotransfer or multiple treatment with m_siRNA 869 alone were sufficient to silence endoglin for up to 60%, but this was not or only slightly reflected in the reduction of tumor growth. On the other hand, a triple electrotransfer of m_siRNA 869 reduced the endoglin mRNA level for up to 80% which resulted in a pronounced antitumor effect and significantly delayed tumor growth. Our results are similar to the results of other *in vivo* studies, where systemic administration of anti-endoglin antibodies was used for the therapy. In contrast to our 3 times local therapy, multiple administrations (4 or 5-times) of antibodies were necessary for the significant effect on tumor growth. In the study of Tsujie et al., significant reduction in tumor growth was obtained in colon-26 and 4T1 tumors after the treatment with anti-endoglin antibodies SN6j; 5 times treatment was used in colon-26 and 4 times in 4T1 breast cancer model [23]. Therefore, our approach, although local, can represent a viable alternative to antibody treatments, especially because it did not result in any changes of animals' weight, indicating that the therapy does not induce systemic toxicity. However, multiple administrations would be required for pronounced antitumor effect. Therefore, this treatment approach, as in the case with antibodies, could be foreseen as an adjuvant treatment to standard chemotherapy or radiotherapy. Furthermore, the systemic delivery of siRNA, which is more acceptable than intratumoral injection is currently accompanied with technical hurdles, as the clearance of siRNA from blood stream is very quick and thus it lacks therapeutic effect [41].

Other antiangiogenic treatment approaches using electrotransfer as a delivery system, utilized siRNAs against Rac1 and VEGF [39], [40]. Rac1 is Rho GTPase involved in VEGF signaling pathway, while endoglin is a TGF-β co-receptor and its expression is highly elevated in tumor blood vessels. Therefore, silencing of endoglin could represent a new approach in antiangiogenic therapies by targeting an alternative signaling pathway to VEGF [42], [43]. In addition, it was shown that TGB-β can stimulate VEGF synthesis in vascular smooth muscle cells, therefore targeting of endoglin might also reduce VEGF in tumors and consequently VEGF-mediated angiogenesis [44]. The *in vivo* results of our study demonstrate that endoglin is an appropriate target as its silencing resulted in a similar antitumor effectiveness as silencing of Rac1. Furthermore, we observed reduced formation of blood vessels in the treated tumors.

In conclusion, the results of our study show that endoglin targeting is a promising antiangiogenic therapy that might represent an alternative to present clinically used anti-VEGF therapies, which are associated with several undesired side effects, such as kidney dysfunction and intracranial hemorrhage [45], [46]. SiRNA molecules showed a good antiangiogenic potential *in vitro* and pronounced antiangiogenic and antitumor effect *in vivo.* Due to the short half-life of siRNA molecules *in vivo*, further studies in this area may continue by using plasmid DNA encoding shRNA or miRNA molecules constructed from the sequence of the siRNA used in our experiments. On the other hand, the stability of siRNA molecules could be also improved by the usage of chemically modified LNA-based oligonucleotides.

## Materials and Methods

### Cell lines, tumors, animals

Human microvascular endothelial cell line HMEC-1 (Centers for Disease Control and Prevention, Atlanta, GA) was cultured in MCDB 131 medium (Gibco, Grand Island, NY) supplemented with 10 μg/l epidermal growth factor (Gibco), 1 mg/l hydrocortisone (Sigma Aldrich, St. Louis, MO), 10% fetal bovine serum (Gibco), 10 mM L-glutamine (Gibco), 50 µg/ml gentamicin (Krka, Novo mesto, Slovenia) and 100 U/ml crystacillin (Pliva d.d, Zagreb, Croatia) in a 5% CO_2_ humidified incubator at 37°C.

Murine endothelial cell line 2H11 (American Type Culture Collection, Manassas, VA) was cultured in Advanced DMEM medium (Gibco) supplemented with 5% fetal bovine serum, 10 mM L-glutamine, 50 µg/ml gentamicin and 100 U/ml crystacillin in a 5% CO2 humidified incubator at 37°C.

Murine mammary adenocarcinoma cells TS/A [47] were cultured in Advanced MEM medium (Gibco) supplemented with 5% fetal bovine serum, 10 mM L-glutamine, 50 µg/ml gentamicin and 100 U/ml crystacillin in a 5% CO_2_ humidified incubator at 37°C.

Female BALB/c mice, 6–8-week old, purchased from Institute of Pathology, Faculty of Medicine, University of Ljubljana, Slovenia were used in the experiments. Mice were maintained under specific pathogen-free conditions at a constant room temperature and humidity and a 12 h light/dark cycle. Food and water were provided *ad libitum*. Animals were subjected to an adaptation period of 2 weeks before experiments.

For induction of subcutaneous tumors, a suspension of 2×10^6^ TS/A cells, prepared from cell culture *in vitro* in 0.1 ml of physiological solution, was injected into the right flank of mice. When the largest diameter of tumors reached 3 mm (in 4–5 days after subcutaneous injection of cells) animals were randomly divided into experimental groups and subjected to a specific experimental protocol.

### Ethics Statement

Animal studies were carried out in accordance with the guidelines for animal experiments of the EU directives and the permission from the Veterinary Administration of Ministry of Agriculture and the Environment of the Republic of Slovenia (permission No.: 34401–12/2009/6). All efforts were made to minimize suffering.

### siRNA molecules

Three siRNA duplexes targeting different sections of coding sequences of human or murine endoglin were chosen on the basis of Invitrogen's program »BLOCK-iT^TM^ RNAi DESIGNER » (Invitrogen, Carlsbad, CA) (Table 3).

**Table 3:** Sequences of siRNA duplexes used in the study.

Against human endoglin	Sense strand (5'–3')	Antisense strand (5'–3')
h_siRNA 529	CGGUGACGGUGAAGGUGGAACUGAG	CUCAGUUCCACCUUCACCGUCACCG
h_siRNA 240	ACGACGCCAUGACCCUGGUACUAAA	UUUAGUACCAGGGUCAUGGCGUCGU
h_siRNA 241	GAGGUGCUUCUGGUCCUCAGUGUAA	UUACACUGAGGACCAGAAGCACCUC

Against murine endoglin	Sense strand (5'–3')	Antisense strand (5'–3')
m_siRNA 868	ACAGCUGCACUCUGGUACAUCUAUU	AAUAGAUGUACCAGAGUGCAGCUGU
m_siRNA 869	CCUGCUGUUUGUCAUCUAUAGCUUU	AAAGCUAUAGAUGACAAACAGCAGG
m_siRNA 150	GAGCUCCCUCUGGUCAGCAAUGUCU	AGACAUUGCUGACCAGAGGGAGCUC

The negative control siRNA duplexes were, by manufacturer's assurance, designed in a way of no homology to any known vertebrate transcript. The siRNA duplexes were obtained as ready-annealed, purified duplexes and diluted in a sterile diethylpyrocarbonate (DEPC) treated H_2_O to a concentration of 20 µM.

### Transfection

#### In vitro lipofection

Cells were trypsinized and harvested from a monolayer to a cell suspension in antibiotics-free medium. Cells (1.4×10^6^) were plated on 24-well ultra-low attachment plate (Corning Inc., Corning, NY) in 1.5 ml of particular medium. Complexes of siRNA duplexes and Lipofectamine RNAiMAX (Invitrogen) were prepared as follows:5 µl of 20 µM siRNA was diluted in 500 μl Opti-MEM I medium (Gibco) to which 5 µl of Lipofectamine RNAiMAX was added. After 15 min incubation of this mixture, the complexes were added to each well. Cells were incubated at 37°C in a 5% CO_2_ humidified incubator for 5 h and then plated on Petri dishes of diameter 10 cm for further analysis: qRT-PCR, immunofluorescence staining, flow cytometry, proliferation assay and tube formation assay.

#### In vivo electrotransfection

*In vivo* electrotransfection was performed once or 3 times on each consecutive day. Tumors were treated with intratumoral injection of 40 µl m_siRNA 869 and immediately thereafter electric pulses were applied (8 square wave pulses of amplitude 240 V (amplitude over distance ratio 600 V/cm), duration 5 ms at frequency 1 Hz) (m_siRNA 869+EP group). Electric pulses were generated by electric pulse generator GT-01 (Faculty of Electrical Engineering, University of Ljubljana, Slovenia) and delivered through 2 parallel stainless steel electrodes with 4 mm distance between them. Due to the relatively large volume of intratumoral injection of m_siRNA 869, the solution also escaped out of the tumor. Therefore, tissues around the tumor that were encompassed between the electrodes were also electrotransfected with m_siRNAs, including tumor surrounding blood vessels, where expression of endoglin is elevated [5]–[7]. Pertinent control groups were: intratumoral injection of H_2_O alone (control group) or combined with application of electric pulses (EP group), intratumoral injection of m_siRNA 869 (m_siRNA 869 group), intratumoral injection of negative control siRNA (siRNA Ctrl group) and electrotransfer of siRNA Ctrl (m_siRNA Ctrl+EP group).

### RNA extraction and qRT-PCR analysis

RNA extraction and qRT-PCR analysis were used for to determine the endoglin expression level and extent of silencing of endoglin at the mRNA level 2 days after transfection *in vitro* and *in vivo*. For *in vitro* experiments, cells were trypsinized and harvested from a monolayer to a cell suspension and then centrifuged. Afterwards the total RNA was extracted from the cells. For *in vivo* experiments, mice were sacrificed and 2 to 9 tumors per group were excised and frozen in liquid nitrogen. Frozen tumors were homogenized in a mortar with a pestle and thereafter the total RNA was extracted from the samples.

The total RNA was extracted with TRIzol Plus RNA Purification System (Invitrogen) according to the manufacturer's instructions. Concentrations of RNA were quantified by a spectrophotometer at 260 nm (Epoch, Biotek, Winooski, VT). The purity of RNA was determined by measuring the ratio of absorbance at A_260 nm/280 nm_ and A_260 nm/230 nm_. One µg of total RNA was reverse transcribed into cDNA using SuperScript VILO^TM^ cDNA Synthesis Kit (Invitrogen), according to the manufacturer's instructions. After reverse transcription, 10×diluted and 100×diluted mixtures were used as a template for the qRT-PCR using TaqMan Gene Expression Master Mix (Applied Biosystems, Carlsbad, CA) and TaqMan Gene Expression Assay (Applied Biosystems). Taqman Gene Expression Assay contained a pair of primers and TaqMan probes to amplify the fragment of human endoglin cDNA (Hs00923996_m1) or murine endoglin cDNA (Mm00468256_m1). As an internal control TaqMan probes were used to amplify human RNA polymerase II cDNA (Hs00172187_m1) or murine 18S ribosomal RNA (Mm03928990_g1). qRT-PCR was performed on 7300 System (Applied Biosystems) as follows: activation of Uracil-DNA Glycosylase (2 min at 50°C), hot start activation of AmpliTaq Gold Enzyme (10 min at 95°C), 45 cycles of denaturation (15 s at 95°C), annealing and extension (1 min 60°C). The qRT-PCR products were analyzed using 7300 System SDS software (Applied Biosystems). The level of endoglin expression in different endothelial and tumor cell lines and TS/A tumors was presented as the threshold cycle value. The level of mRNA of endoglin after therapy was normalized against the reference genes.

### Immunofluorescence staining

Two days after the transfection, the extent of silencing of endoglin at the protein level was determined by immunofluorescence staining. Cells were trypsinized and 5×10^4^ cells were plated on Lab-Tek Chamber Slide System (Nalge Nunc International, Naperville, IL) in an appropriate medium containing 10% fetal bovine serum and antibiotics. Attached cells were fixed in 4% paraformaldehyde (Electron Microscopy Sciences, Hatfield, PA) for 15 min. Fixed cells were then permeabilized with 0.5% Tween 20 (Sigma) for 10 min. Nonspecific binding was blocked with 10% donkey serum (Sigma). Afterwards, cells were incubated with primary antibodies overnight at 4°C. For HMEC-1 cells, mouse monoclonal antibodies against endoglin were used (P3D1: sc-18838, dilution 1∶50, Santa Cruz Biotechnology Inc., Santa Cruz, CA). After incubation with primary antibodies, cells were incubated with fluorescein-conjugated donkey anti-mouse secondary antibodies for 1 h (1∶100, Jackson ImmunoResearch Laboratories, Inc., West Grove, PA). For 2H11 cells, goat polyclonal antibodies against endoglin were used (AF1320, dilution 1∶12.5, R&D Systems Inc., Minneapolis, MN). After incubation with primary antibodies, cells were incubated with fluorescein-conjugated donkey anti-goat secondary antibodies for 1 h (1∶100, Jackson ImmunoResearch Laboratories, Inc.). Between each step, the cells were washed 3-times with phosphate buffered saline. After incubation with secondary antibodies, slides were mounted with Prolong Gold Antifade Reagent (Invitrogen). Images were captured with a DP72 CCD camera (Olympus, Hamburg, Germany) connected to a BX-51 microscope (Olympus). The exposure time and acquisition settings were determined on control cells and kept constant throughout the aquisition.

### Flow cytometry

For quantification of silencing of endoglin at the protein level, flow cytometry analysis was performed. Two days after transfection, cells were trypsinized and 1×10^6^ cells were resuspended in phosphate buffered saline. Cells were fixed in 4% paraformaldehyde for 15 min and permeabilized with 0.5% Tween 20 for 10 min. The HMEC-1 cells were then incubated with primary fluorescein-conjugated mouse monoclonal antibodies (P3D1 FITC: sc-18838, 1∶50, Santa Cruz Biotechnology, Inc.) overnight at 4°C. The next day, primary antibody solution was removed and cells were resuspended in 300 µl of phosphate buffered saline. Mouse 2H11 cells were after permeabilization first incubated with primary goat anti-mouse polyclonal antibodies (1∶50) overnight at 4°C. The next day, cells were incubated with fluorescein-conjugated donkey anti-goat secondary antibodies for 1 h (1∶100). Between each step, the cells were washed 3-times with phosphate buffered saline. The measurements were performed on at least 20.000 cells per sample using FACSCanto II flow cytometer (BD Biosciences, San Jose, CA). A 488-nm laser (air-cooled, 20 mW solid state) and 530/30-nm band-pass filter were used for the excitation and detection of FITC fluorescence, respectively. Firstly, the cell population gate was determined from the biparametric logarithmic plot defined by forward and side scatter to eliminate debris. Secondly, the histogram of gated cells against their fluorescence intensity was recorded. The mean fluorescence intensity of the gated cells was determined for each experimental group (software: BD FACSDiva V6.1.2).

### Proliferation assay

After the transfection, 1.5×10^3^ HMEC-1, 3×10^2^ 2H11 cells or 2×10^2^ TS/A cells were plated for proliferation assay in 0.1 ml of particular media on 96-well plates (Corning) containing 10% fetal bovine serum and antibiotics. Cells were incubated at 37°C in a 5% CO_2_ humidified incubator. Cell viability was measured every second day with Presto Blue assay (Invitrogen). Ten µl of Presto Blue reagent was added to each well and fluorescence intensity in the well was measured after 1.5 h of incubation at 37°C in a 5% CO_2_ humidified incubator with a microplate reader (Infinite 200, Tecan, Männedorf, Switzerland).

### Tube formation assay

To determine the effect of silencing of endoglin on endothelial cell tube formation, cells were incubated at 37°C in a 5% CO_2_ humidified incubator for different period of time after the transfection. At days 1, 2 and 3 after transfection, 1.5×10^4^ HMEC-1 cells were plated on 96-well plates (Corning) covered with BD Matrigel^TM^ Basement Membrane Matrix Growth Factor Reduced, Phenol Red Free (BD Biosciences) and incubated for 6 h until the formation of tubular complexes. The tubular complexes were fixed and stained with Crystal violet dye (Sigma). For assessment of tube formation of 2H11 cells, an improved system for assessment of tube formation was used [48]. Cells (1.6×10^4^) were plated two days after the transfection on µ-Slide Angiogenesis (Ibidi, Munich, Germany) covered with BD Matrigel^TM^ Basement Membrane Matrix, Phenol Red Free (BD Biosciences) and incubated for 2 h until the formation of tubular complexes. The tubular complexes were stained with calcein AM (Sigma). Images were captured with a DP72 CCD camera (Olympus) connected to an IX-70 inverted microscope (Olympus). AxioVision program (Carl Zeiss Microscopy GmbH, Jena, Germany) was used to convert raw images into binary masks, which were quantified with AngioQuant image analysis program [49]. The total length of tubular complexes, the total size of tubular complexes and the total number of junctions were quantified.

### Tumor growth

The therapeutic potential of electrotransfer of m_siRNA 869 molecules against murine endoglin was assessed by measuring the tumor size every day after the therapy using digital Vernier caliper. Tumor volume was calculated according to the formula for ellipsoid: V = a×b×c× π/6, where a, b and c represent perpendicular tumor diameters. From the tumor growth curves, the tumor tripling time (the time in which the tumor volume at the start of the treatment triplicates) was determined. The weight of the mice was followed as a general index of systemic toxicity. Each group consisted of 6 mice.

### Histology

From each experimental group, 2 or 3 mice were sacrificed at day 2 post-treatment and the tumors were excised. The tumors were fixed in IHC zinc fixative (BD Pharmingen, BD Biosciences) and embedded in paraffin. Three consecutive 5-μm thick sections were cut from each paraffin block. The first section was stained with hematoxylin and eosin and the second and the third were used for immunohistochemical staining. The immunohistochemical staining sections were incubated with rabbit polyclonal antibodies against murine CD31 (ab28364, Abcam, Cambridge, MA) at dilution 1∶100 or Ki-67 (clone SP6, Thermo Fisher Scientific, Cambridge, MA) at dilution 1∶150. A peroxidase-conjugated streptavidin–biotin system (Rabbit specific HRP/DAB detection IHC kit, ab64261, Abcam) was used as the colorogenic reagent followed by hematoxylin counterstaining. The immunohistochemicaly stained slides were observed under light microscopy and from each slide 6 images of randomly selected areas of viable tumor tissue were captured with a DP72 CCD camera (Olympus) connected to a BX-51 microscope (Olympus). On the acquired images the number of Ki-67 positive cells and CD31 positive blood vessels with the diameter of less than 30 µm was determined.

### Statistical analysis

All data were tested for normality of distribution with the Shapiro-Wilk test. The differences between the experimental groups were statistically evaluated by one-way analysis of variance (one-way ANOVA) followed by a Holm-Sidak test for multiple comparison. A P-value of less than 0.05 was considered to be statistically significant. SigmaPlot Software (Systat Software, Chicago, IL) was used for statistical analysis and graphical representation.

## Supporting Information

Table S1 **Tripling time of TS/A tumors treated with triple electrotransfer of siRNA against endoglin.** After repetitive treatment of tumors with m_siRNA 869 a statistically significant tumor growth delay was observed at the level of tumor tripling time (related to Figure 4).
(DOC)
