# Supplementary material for: Multiple Delivery of siRNA against Endoglin into Murine Mammary Adenocarcinoma Prevents Angiogenesis and Delays Tumor Growth
Source: PLoS One. 2013 Mar 5;8(3):e58723. doi: 10.1371/journal.pone.0058723 (PMC3589348; doi:10.1371/journal.pone.0058723)
Supplement: Table S1 — Tripling time of TS/A tumors treated with triple electrotransfer of siRNA against endoglin. After repetitive treatment of tumors with m_siRNA 869 a statistically significant tumor growth delay was observed at the level of tumor tripling time (related to Figure 4). (DOC) [file pone.0058723.s001.doc]

Table S1.

| Group | n | Tripling time (days) |
| --- | --- | --- |
| Control | 6 | 0.86 ± 0.14 |
| EP | 6 | 0.94 ± 0.17 |
| m_siRNA 869 | 6 | 1.21 ± 0.09 |
| m_siRNA 869 + EP | 6 | 3.23 ± 0.33 * |
| siRNA Ctrl | 6 | 0.92 ± 0.15 |
| siRNA Ctrl + EP | 6 | 0.84 ±0.13 |

Data represent arithmetic mean ± SEM; *P<0.001 compared to all other groups
